# Necroptosis Stimulates Interferon-Mediated Protective Anti-Tumor Immunity

**DOI:** 10.21203/rs.3.rs-3713558/v1

**Published:** 2023-12-19

**Authors:** Francis Chan, A. Justin Rucker, Christa Park, Qi-Jing Li, E Ashley Moseman

**Affiliations:** Liangzhu Laboratory, Zhejiang University; Duke University; University of Massachusetts Chan Medical School; Agency for Science, Technology and Research (A*STAR); Duke University

## Abstract

Necroptosis is an inflammatory form of cell suicide that critically depends on the kinase activity of Receptor Interacting Protein Kinase 3 (RIPK3). Previous studies showed that immunization with necroptotic cells conferred protection against subsequent tumor challenge. Since RIPK3 can also promote apoptosis and NF-κB-dependent inflammation, it remains difficult to determine the contribution of necroptosis-associated release of damage-associated molecular patterns (DAMPs) in anti-tumor immunity. Here, we describe a system that allows us to selectively induce RIPK3-dependent necroptosis or apoptosis with minimal NF-κB-dependent inflammatory cytokine expression. In a syngeneic tumor challenge model, immunization with necroptotic cells conferred superior protection against subsequent tumor challenge. Surprisingly, this protective effect required CD4^+^ T cells rather than CD8^+^ T cells and is dependent on host type I interferon signaling. Our results provide evidence that death-dependent type I interferon production following necroptosis is sufficient to elicit protective anti-tumor immunity.

## Introduction

Necroptosis is widely viewed as an inflammatory form of cell death due to the release of DAMPs from ruptured plasma membrane. Receptor interacting protein kinase 3 (RIPK3), the essential serine/threonine kinase in necroptosis, can be activated by one of three distinct upstream activators: the related kinase RIPK1, the toll-like receptor 3 (TLR3) and TLR4 adaptor TIR domain containing adaptor molecule 1 (TRIF), and the viral RNA sensor Z-DNA binding protein 1 (ZBP1). RIPK3 oligomerizes and forms cytosolic signaling complexes via its RIP homotypic interaction motif (RHIM), which allows it to phosphorylate the necroptosis effector molecule Mixed Lineage Kinase domain-Like (MLKL). Phosphorylation causes MLKL to oligomerize and translocate to the plasma membrane to induce membrane rupture, leading to the leakage of damage associated molecular patterns (DAMPs) and inflammatory responses (1, 2). In addition to promoting necroptosis, RIPK3 can also participate in apoptosis in certain situations. For instance, in the absence of MLKL or when the kinase activity of RIPK3 is inhibited, RIPK3 can stimulate formation of an alternate apoptosis-inducing complex with caspase-8, RIPK1, and FAS-associated death domain protein (FADD) (3). Moreover, RIPK3 can also stimulate inflammatory gene expression in a RHIM-dependent but cell death-independent manner (4–7). In this context, the RHIM serves as a scaffold to stimulate NF-κB activation (4, 8, 9).

RIPK3 expression is downregulated in many tumor types, suggesting that RIPK3 has important functions in tumor suppression (10, 11). In support of its anti-tumor role, lower RIPK3 expression correlates with worsened patient survival in lung cancer (12), chronic lymphocytic leukemia (13), colon cancer (14), malignant mesothelioma (15), and breast cancer (16). In contrast, tumor RIPK3 expression appears to be beneficial for tumor immune surveillance. For instance, expression of RIPK3 and other necroptotic adaptors in tumor cells was associated with improvement in CD8^+^ T cell infiltration in hepatocellular carcinoma (17), cholangiocarcinoma (18), and prostate cancer (19).

The current gold standard approach for studying the immunogenicity of cell death *in vivo* is to use dead or dying cells as a tumor vaccine to immunize syngeneic mice (20). Previous studies have demonstrated that RIPK3-induced necroptosis promotes dendritic cell (DC) cross-priming of tumor-specific CD8^+^ T cells to achieve control of tumor growth. However, necroptosis was accompanied by strong NF-κB-dependent cytokine expression in these studies. Thus, it was not possible to distinguish the direct contribution of necroptosis-induced DAMPs release in the induction of anti-tumor immunity (21–24). Further, cell-intrinsic NF-κB signaling during necroptosis can also promote carcinogenesis (25). Consequently, there remains a need to clarify the consequences of these distinct aspects of tumor RIPK3 signaling in the antitumor response.

We previously showed that in a doxycycline (DOX)-inducible system, RIPK3 expression accompanied by proteasome inhibition was sufficient to drive necroptosis in 3T3 fibroblasts. Here, we adopted this DOX-inducible system in tumor cells. In contrast to other models in which RIPK3 activation was achieved using chemical-induced dimerization of synthetic RIPK3 chimeric cassettes (3), RIPK3 activation in our system was independent of RIPK1 and did not induce strong NFκB activation. By selectively restricting cell death to either RIPK3-dependent apoptosis or necroptosis, we found that immunization with necroptotic cells, but not apoptotic cells showed marked protection to subsequent tumor challenge. Surprisingly, immunization with necroptotic cells stimulated an anti-tumor CD4^+^ T cell response while CD8^+^ T cells were dispensable for tumor protection. The protection conferred by necroptotic cell immunization was observed with tumors from different tissue origin. Mechanistically, we showed that interferon beta (IFNβ) was specifically induced in necroptosis but not in apoptosis, and blocking cell death effectively eliminated this type I IFN response. Furthermore, the protective effect of necroptosis immunization was abrogated when host IFN signaling was inhibited by IFNAR deficiency. These data suggest that necroptosis in the absence of NF-κB dependent cytokine expression drives anti-tumor immunity through a distinct type I IFN and CD4^+^ T cell dependent mechanism.

## Results

### Expression of RIPK3 accompanied by proteasome inhibition drives tumor cell necroptosis

**Figure 1:**
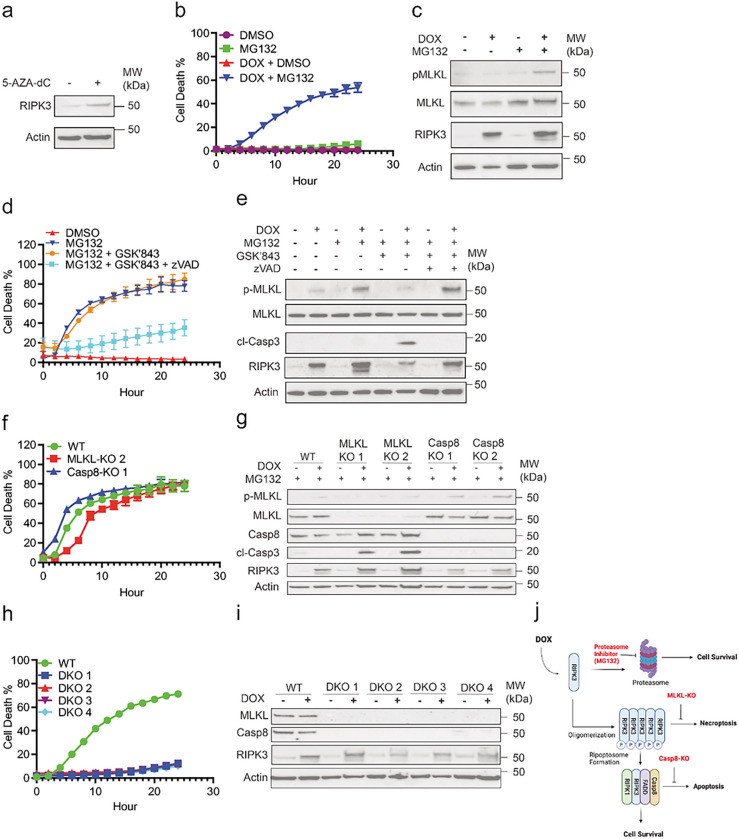
RIPK3-expressing tumor cells undergo necroptosis or RIPK3-dependent apoptosis upon proteasome inhibition. **(a)**LLC-OVA cells were treated with DMSO or 5-AZA-dC (30 mM) for 72 hours prior to lysing cells for western blot. Data is representative of two independent experiments. **(b-c)** Transduced LLC-OVA cells (WT) were treated with DMSO or DOX (1 mg/mL) for 8 hours prior to treatment with either DMSO or MG132 (4 mM). **(b).** Cell death was measured by tracking YoYo1 (50 nM) uptake in cells via Incucyte. **(c)** Cell lysates were collected 4 hours after treatment with MG132 for western blot. For **b-c**, data is representative of greater than three independent experiments. **(d-e)** WT cells were treated with DMSO or DOX (1 mg/mL) for 7 hours, cells were pre-treated with ZVAD (20 mM) 30 mins prior to GSK’843 (20 mM) then MG132 (4 mM) after another 30 mins. **(d)** Cell death was monitored via Incucyte. **(e)** Cell lysates were collected 4 hours after MG132 treatment for western blot. For **d-e**, data is representative of two independent experiments. **(f-g)** WT, Casp8-KO, and MLKL-KO LLC-OVA cells were treated for 8 hours with DOX (1 mg/mL) followed by treatment with MG132 (4 mM). **(f)**Cell death measured via Incucyte. **(g)** Cell lysates were collected at 4 hours following treatment with MG132 for western blot. For **f-g**, data is representative of two independent experiments. **(h-i)** WT or MLKL- and Casp8-KO (DKO) cells were treated for 8 hours with DOX (1 mg/mL) followed by treatment with MG132 (4 mM). **(h)** Cell death measured via Incucyte. **(i)** Cell lysates were collected at 4 hours following treatment with MG132 for western blot. For **h-i**, data is from a single experiment. **(j)** Graphical summary of RIPK3-dependent cell death in tumor cells following proteasome inhibition. Image was created with BioRender.com.

RIPK3 expression is frequently inhibited in tumor cells through promoter hypermethylation (26). To explore whether re-expression of RIPK3 might enhance anti-tumor immune surveillance, we first attempted to restore expression of endogenous RIPK3 in Lewis Lung Carcinoma cells expressing chicken ovalbumin (LLC-OVA) using the DNA methyltransferase inhibitor 5-AZA-dC. Although 5-AZA-dC successfully restored RIPK3 expression, its toxicity prevented further exploration of cell death responses (Fig. 1a **and data not shown)**. We therefore opted to utilize a doxycycline (DOX)-inducible system to restore RIPK3 expression in LLC-OVA cells. Consistent with previous report (3), DOX-induced expression of RIPK3 was not sufficient to cause cell death of the LLC-OVA cells. However, addition of the proteasome inhibitor MG132 to prevent RIPK3 degradation led to strong cell death in DOX-induced LLC-OVA cells (Fig. 1b). This cell death was dependent on RIPK3 expression, since MG132 alone did not compromise cell survival. Increased phosphorylation of MLKL (pMLKL) was observed in DOX- and MG132-treated cells, indicating that necroptosis was the dominant form of cell death (Fig. 1c). The RIPK3 kinase inhibitor GSK’843 did not inhibit cell death (Fig. 1d), although it effectively inhibited pMLKL (Fig. 1e). Rather, GSK’843 increased caspase-3 cleavage (Fig. 1e), suggesting a switch from necroptosis to apoptosis (6). Indeed, co-treatment with GSK’843 and the pan-caspase inhibitor zVAD-fmk largely abrogated pMLKL, caspase-3 cleavage and cell death (Fig. 1d–e). Since pMLKL and caspase-3 cleavage required DOX-induced RIPK3 expression (Fig. 1e), these results indicate that RIPK3 has the capacity to promote necroptosis as well as apoptosis.

### Selective induction of RIPK3-dependent tumor cell necroptosis or apoptosis

The switch from necroptosis to apoptosis with RIPK3 kinase inhibitor revealed a possible method to manipulate RIPK3-dependent cell death. To circumvent the off-target effects of chemical inhibitors, we utilized the CRISPR/Cas9 system to inactivate either *caspase-8* (Casp8) or *Mlkl* in the DOX-inducible LLC-OVA cells (WT). Casp8-KO and MLKL-KO cells underwent DOX- and MG132-induced cell death with similar kinetics and magnitude when compared to WT cells (Fig. 1f). As in the case of RIPK3 kinase inhibition, MLKL-KO cells exhibited increased caspase-3 cleavage, indicating a switch from necroptosis to apoptosis (Fig. 1g). By contrast, Casp8-KO retained pMLKL but did not exhibit any caspase-3 cleavage (Fig. 1g). Knockout of both *Mlkl* and *Casp8* completely abrogated cell death (Fig. 1h–i). Hence, selective activation of RIPK3-dependent necroptosis and apoptosis was achieved by genetic inactivation of *Casp8* and *Mlkl* respectively (Fig. 1j).

### RIPK3-induced tumor cell death does not elicit strong cytokine response

**Figure 2:**
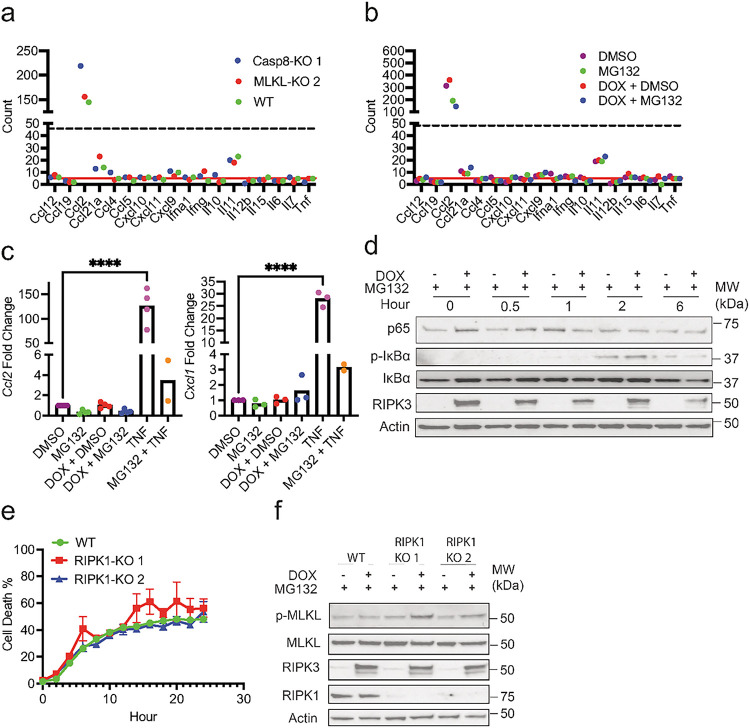
Cell death following proteasome inhibition occurs in the absence of RIPK1 and NFkB-dependent cytokine production. **(a-b)** WT, Casp8-KO, and MLKL-KO LLC-OVA cells were treated for 8 hours with DOX (1 mg/mL) followed by treatment with MG132 (4 mM). Four hours after treatment with MG132, RNA was prepped using tumor cell lysates. Tumor cytokine expression was measure via Nanostring using the mouse tumor 360 signaling panel. **(a)** Cytokine production in tumor cells with DOX + MG132. **(b)** Tumor cytokine production in WT cells with DMSO, MG132, DOX + DMSO, or DOX + MG132. For **a-b**, data is from a single experiment. The red solid line indicates the mean read count for the negative controls and the dashed black line indicates the mean read count for the lowest positive control. **(c-d)** WT cells were treated in a similar fashion to **a-b**. Four hours after treatment with MG132, RNA was prepared from tumor cell lysates for qPCR. **(c)** Gene expression for *Ccl2* and *Cxcl1* following treatments as indicated. Each point represents an average of technical replicates from an individual experiment. **(d)** WT cells were treated with DMSO or DOX (1 mg/mL) 8 hours prior to treatment with MG132 (4 mM). Cell lysates were collected immediately, 30 mins, 1 hour, 2 hours, and 6 hours after treatment with MG132 for western blot. Data is representative of two independent experiments. **(e-f)** RIPK1 knockout in WT cells was performed using the CRISPR/Cas9 system. RIPK1 KO and WT cells were then treated with MG132 (4 mM) following an 8-hour induction with DOX (1 mg/mL). **(e)** Lysates were collected 4 hours following treatment with MG132 for western blot. Data is a from a single experiment. **(f)** Cell death was measured using Incucyte. Data is representative of two independent experiments. For **c**, treatment groups were compared using two-way ANOVA. *P < 0.05, **P < 0.01, ***P < 0.001, and ****P < 0.0001.

Several studies have shown that chemical-induced dimerization of RIPK3 concomitantly led to necroptosis and strong RIPK1-mediated, NFκB-dependent cytokine expression (22, 23). In contrast to these studies, Nanostring analysis revealed that cytokine expression was largely undetectable in DOX- and MG132-treated WT, Casp8-KO, and MLKL-KO LLC-OVA cells (Fig. 2a–b). While *Ccl2* was the only detectable cytokine in the Nanostring panel (Fig. 2a), its expression was not enhanced by RIPK3 expression nor cell death (Fig. 2b; **Supplemental Fig. 1)**. In fact, qPCR analysis revealed that MG132 modestly reduced *Ccl2* expression (Fig. 2c).

Yatim and colleagues found that chemical-induced RIPK3 dimerization led to RHIM-dependent recruitment and activation of RIPK1 and cytokine expression (22). However, NF-κB activation as determined by IκBα phosphorylation (p-IκBα) and IκBα degradation was minimal and independent of RIPK3 expression (Fig. 2d). CRISPR/Cas9 knockout of *Ripk1* (RIPK1-KO) did not affect DOX- and MG132-induced cell death (Fig. 2e–f), indicating that necroptosis in our LLC-OVA cells was RIPK1-independent. Taken together, these data indicate that RIPK1- and NF-κB-dependent cytokine production was absent in our necroptosis induction system.

### Immunization with necroptotic cells protects against tumor challenge

**Figure 3:**
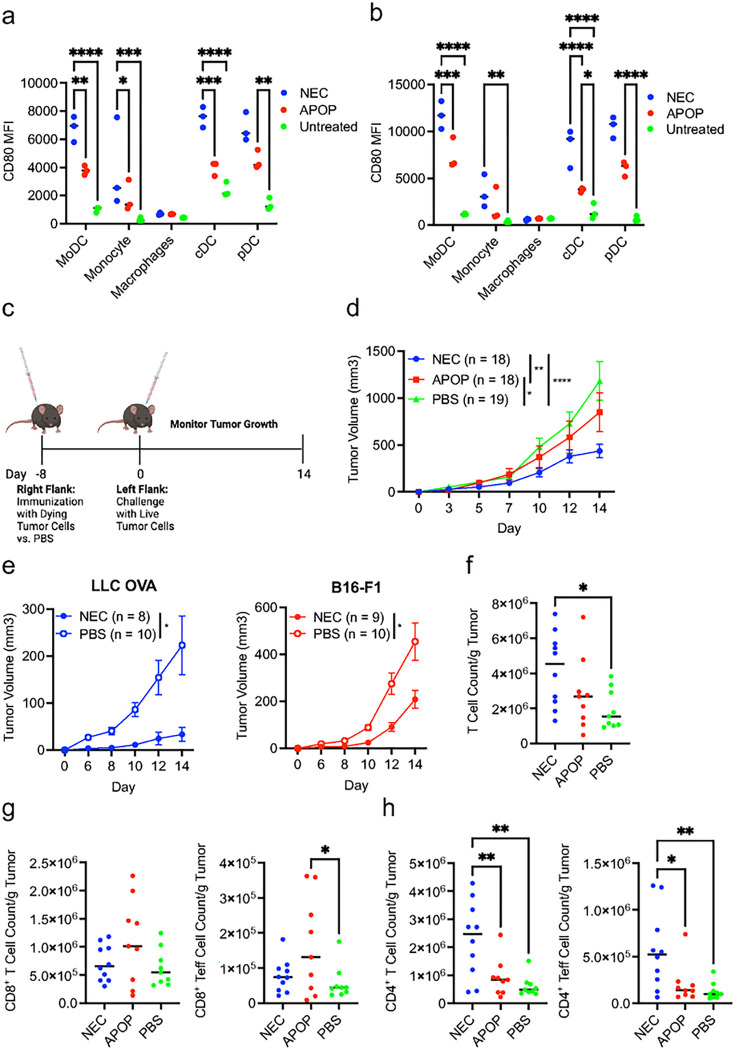
Necroptotic dying cells stimulate superior protection against tumor challenge than apoptotic dying cells. **(a-b)** Splenocytes from B16-Flt3L tumor bearing mice were co-cultured with dying cells. CD80 Mean Fluorescence Intensity (MFI) was then assessed on splenic myeloid cells by flow cytometry at 8 hours **(a)** and 24 hours **(b)** post-initiation of co-culture. Data is representative of two independent experiments (n = 3 per experiment). **(c)** Schematic of experimental model for assessing anti-tumor immune response following dying cell immunization. Image was created with BioRender.com. **(d-g)** NEC, APOP or PBS was injected into the right flank of mice 8 days prior to challenge with live LLC-OVA cells. **(d)** Tumor volume was assessed. Plot represents aggregated data from 4 independent experiments (n = 4 – 5 per treatment group per experiment). **(e)** Mice were immunized with NEC LLC-OVA or PBS followed by challenge with live LLC-OVA or B16-F1 melanoma as indicated. **(f-h)** Tumors were harvested at day 10 post-tumor challenge to assess for tumor infiltration by total **(f)** T cells, **(g)** CD8^+^ T cells and Teff cells (CD44^Hi^CD62L^−^), and **(h)** CD4^+^ T cells and Teff cells per gram of tumor via flow cytometry. Data aggregated from two independent experiments (n = 4 – 5 per treatment group per experiment). For **a-b and e-h**, treatment groups were compared using one-way ANOVA. For **d,** treatment groups were compared using two-way ANOVA. *P < 0.05, **P < 0.01, ***P < 0.001, and ****P < 0.0001.

Immunogenic cell death (ICD) such as necroptosis was widely thought to stimulate immune responses through the release of DAMPs. We tested this premise using our LLC-OVA cell lines. Casp8-KO or MLKL-KO LLC-OVA cells were treated with DOX and MG132 to induce necroptosis and apoptosis respectively, and co-cultured with splenic DCs from Flt3L-treated mice. We found that necroptotic cells (NEC) bolstered expression of the costimulatory molecule CD80 on multiple DC subsets and monocytes compared to either apoptotic cells (APOP) or untreated controls (Fig. 3a–b, **Supplemental Fig. 2)**, consistent with the notion that necroptosis is more immunogenic than apoptosis.

We next immunized mice with NEC or APOP subcutaneously followed by challenge with live LLC-OVA cells on the opposite flank eight days post-immunization (Fig. 3c). Importantly, when compared to PBS or APOP-immunized groups, tumor growth was significantly blunted by NEC immunization (Fig. 3d). NEC immunization similarly protected the hosts from subsequent challenge with B16-F1 melanoma (Fig. 3e). Hence, NEC immunization provides protection against tumors of different tissue origin. NEC immunization increased overall T cell infiltration in the tumor (Fig. 3f). In contrast to previous studies in which necroptosis was shown to be superior in promoting CD8^+^ T cell responses (21–23), effector/memory CD8^+^ T cell (CD8^+^CD44^hi^CD62L^−^) infiltration was similar between NEC and APOP immunization groups (Fig. 3g). By contrast, overall CD4^+^ T cell and effector CD4^+^CD44^hi^CD62L^−^ T cell infiltration was significantly elevated in NEC-immunized mice compared to APOP-immunized or PBS-treated mice (Fig. 3h).

### Necroptosis immunization stimulates CD4^+^ T cell-dependent anti-tumor immunity

**Figure 4:**
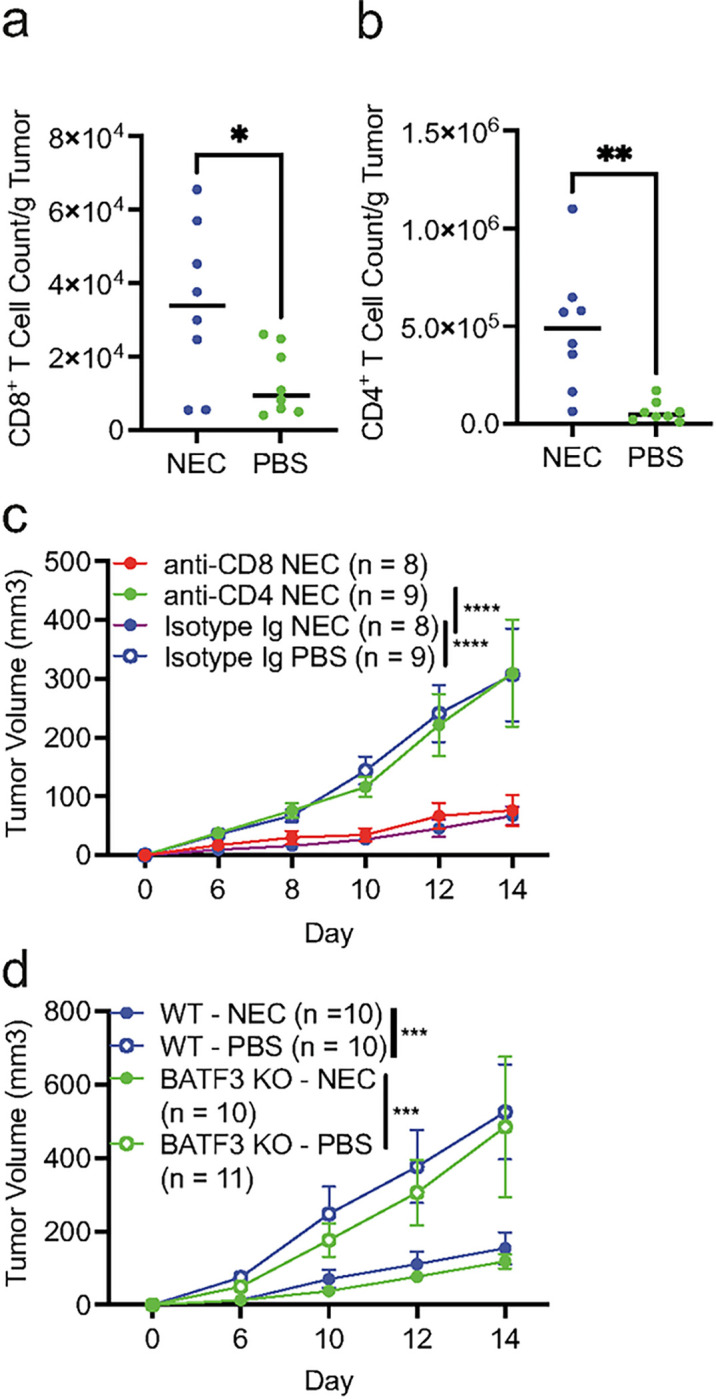
Tumor restriction with necroptotic dying cell immunization is CD4+ T cell dependent. **(a-b)** Quantification of total numbers of **(a)** CD8^+^ T cells and **(b)** CD4^+^ T cells per gram of tumor from tumors of NEC or PBS immunized mice at day 5 post tumor challenge. Treatment groups were compared using unpaired Student’s t-test. Data is aggregated from two independent experiments (n = 4 – 5 per treatment group per experiment). **(c)** Mice received anti-CD8a, anti-CD4, or Isotype control antibody prior to immunization with NEC or PBS followed 8 days later by challenge with live tumor cells. Data is aggregated from two independent experiments (n = 4 – 5 per treatment group per experiment). **(d)** NEC immunization and subsequent live tumor challenge was performed in WT or *Batf3*^−/−^ mice with tumor volume assessment at the indicated timepoints. Data is aggregated from two independent experiments (n = 5 – 6 per treatment group per experiment). For **c-d**, treatment groups were compared using two-way ANOVA. For **f**, treatment groups were compared using one-way ANOVA. *P < 0.05, **P < 0.01, ***P < 0.001, and ****P < 0.0001

Increase in CD8^+^ and CD4^+^ T cell infiltration in NEC-immunized mice in comparison to PBS control group was already evident on day 5 post-implantation when tumors were first palpable (Fig. 4a–b). This is in contrast to tumor myeloid populations, which were comparable at this timepoint **(Supplemental Fig. 3)**. To test the contribution of CD4^+^ and CD8^+^ T cells in the protective effect conferred by NEC immunization, we used antibodies to deplete these populations. Importantly, only the depletion of CD4^+^ cells, but not CD8^+^ cells prior to immunization was able to abrogate the tumor protection by NEC immunization (Fig. 4c). Consistent with the dispensable role of CD8^+^ T cell in this process, disrupting cDC1-dependent cross-priming of CD8^+^ T cells using *Batf3*-deficient hosts did not affect the tumor suppressive effect of NEC immunization (Fig. 4d). In aggregate, these data suggest that NEC immunization confers anti-tumor restriction through CD4^+^ T cells and independent of CD8^+^ T cells.

### Full CD4^+^ T cell priming requires secondary challenge with live tumor cells

**Figure 5:**
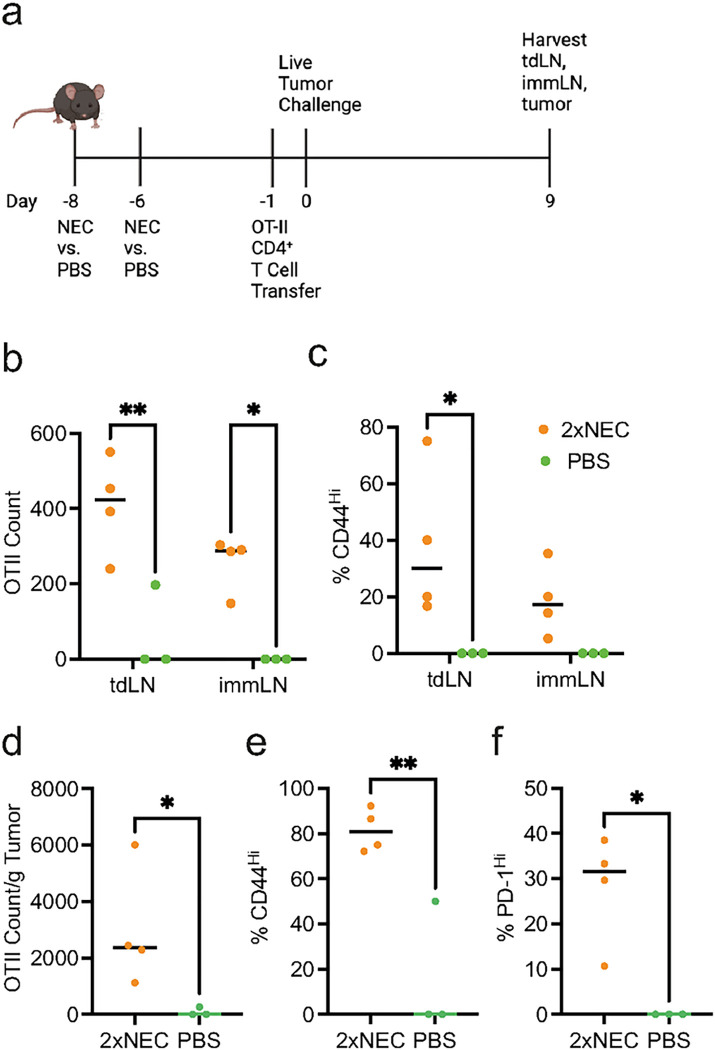
Necroptotic dying cell immunization results in dying cell antigen-independent tumor suppression. **(a)** Mice were immunized with NEC and boosted with a second immunization two days later (2x NEC). Mice subsequently received OT-II cells five days later followed by challenge with live tumor challenge the next day. At day 9 post-tumor implant the tumor and draining inguinal lymph nodes from the tumor (tdLN) and immunization site (immLN) were collected for flow cytometry. Image was created with BioRender.com. **(b)** Total count and **(c)** % of CD44^Hi^ for OT-II CD4^+^ T cells found in the lymph nodes as indicated. Treatment groups were compared using two-way ANOVA. **(d)** Total count per gram of tumor, **(e)** % of CD44^Hi^ and **(f)** % of PD-1^Hi^ for tumor OT-II CD4^+^ T cells. Treatment groups were compared using unpaired Student’s t-test. For **a-f**, data is representative of two independent experiments (n = 3 – 4 per treatment group per experiment). Plot represents aggregated data from two independent experiments (n = 4 – 5 per treatment group per experiment). Treatment groups were compared using two-way ANOVA. *P < 0.05, **P < 0.01, ***P < 0.001, and ****P < 0.0001.

To test whether CD4^+^ T cell priming occurs during the immunization phase, we adoptively transferred CD4^+^ T cells from OT-II mice and found that NEC immunization did not enhance OT-II CD4^+^ T cells accumulation in the draining lymph node (**Supplemental Fig. 4a)**, even after a second immunization with NEC cells **(Supplemental Fig. 4b-c)**. In contrast, increased CD4^+^ OT-II T cells accumulation in response to NEC immunization was detected in the draining lymph nodes (Fig. 5b–c) and tumor (Fig. 5d–f) when OT-II T cells were adoptively transferred the day prior to tumor challenge (Fig. 5a). This effect was evident whether the host received a single or multiple doses of NEC immunization **(Supplemental Fig. 4d-e)**.

### Type I interferon mediates protection conferred by necroptosis immunization

**Figure 6:**
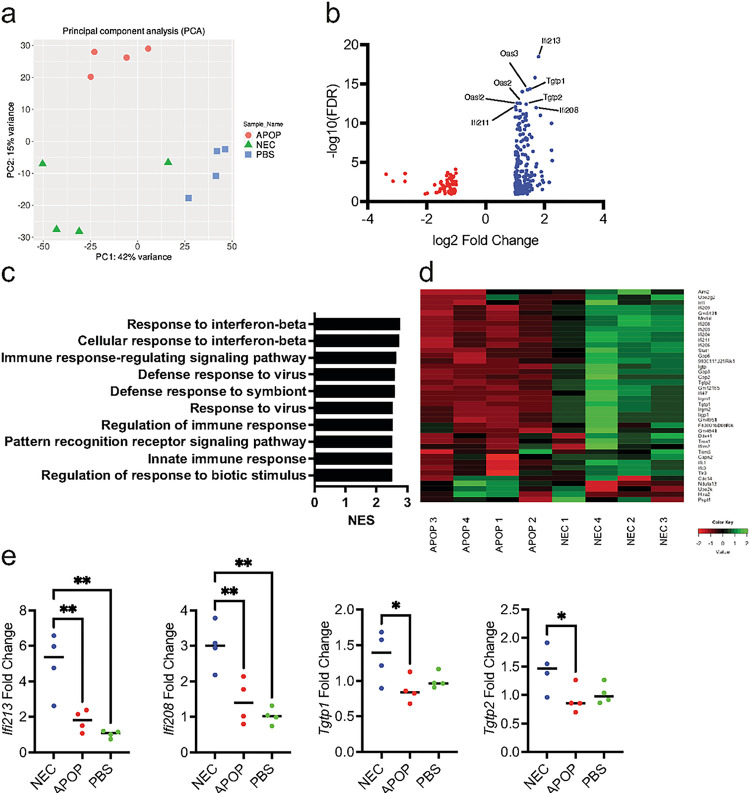
Immunization with necroptotic dying cells induces a type I interferon gene signature in the tumor. **(a-d)**Mice were immunized with NEC, APOP, or PBS followed 8 days later by challenge with live tumor cells. At day 14 post-tumor challenge, tumors were harvested, and RNA was prepared from tumor single cell suspensions. Bulk RNA-seq was subsequently performed. **(a)** Principal component analysis of tumor samples. **(b)** Top differentially expressed genes for NEC- vs. APOP-immunized tumors. **(c)** GSEA pathway analysis for NEC- vs. APOP-immunized tumors. **(d)** Heatmap for “Response to interferon beta” GO term in NEC- and APOP-immunized tumor samples. **(e)** Tumor RNA was used for qPCR using primers for the indicated ISGs. Data is from a single experiment (n = 4 per treatment group). Treatment groups were compared using one-way ANOVA. *P < 0.05, **P < 0.01, ***P < 0.001, and ****P < 0.0001.

To interrogate the mechanism by which NEC immunization conferred protection against tumor challenge, we performed bulk RNA sequencing on the tumor tissues. We found that the gene expression largely clustered based on the different immunization regimen (Fig. 6a). In comparing the top differentially expressed genes between the NEC- and APOP-immunized groups, we found that the majority were interferon stimulated genes (ISGs) (Fig. 6b). Gene Set Enrichment Analysis (GSEA) further confirmed that NEC-immunized tumors showed enrichment for genes involved in response to interferon-beta (IFN-β) (Fig. 6c–d). Increased expression of several ISGs in tumors from NEC-immunized mice compared to those from APOP-immunized mice was further validated by qPCR (Fig. 6e).

**Figure 7:**
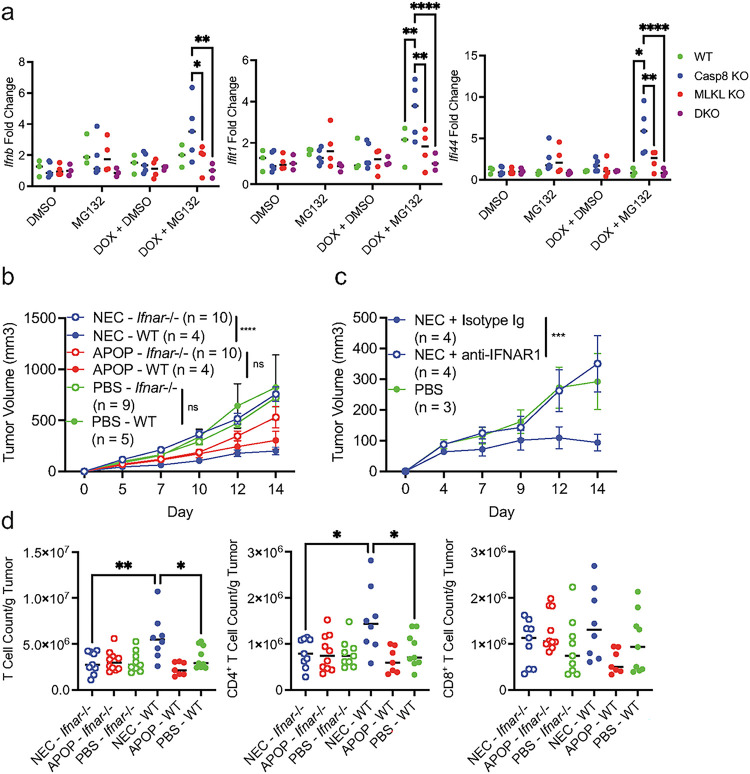
Anti-tumor immunity induced by necroptotic dying cells is abrogated with loss of type I interferon signaling. **(a)** WT, Casp8-KO, MLKL-KO, and DKO LLC-OVA cells were treated for 8 hours with DOX (1 mg/mL) followed by treatment with MG132 (4 mM). Four hours after treatment with MG132, RNA was prepped using tumor cell lysates. Gene expression for *Ifnb*, *Ifi44*, and *Ifit1* was assessed by qPCR as indicated Each point represents an average of technical replicates from an individual experiment. **(b)** Dying cell immunization and subsequent live tumor challenge was performed in WT or *Ifnar1*^−/−^ mice. Data is aggregated from two independent experiments (n = 4 – 5 per treatment group per experiment). **(c)** Anti-IFNAR1 antibody or Isotype control was administered the day prior to necroptotic cell immunization. Live tumor challenge was performed 8 days post-immunization. Data is representative of three independent experiments (n = 3 – 5 per treatment group per experiment). **(d)** T cell infiltrate was assessed in tumors at day 14 post-tumor challenge in in WT or *Ifnar1*^−/−^ mice. Data is aggregated from two independent experiments (n = 4 – 5 per treatment group per experiment). For **a & d,** treatment groups were compared using one-way ANOVA. For **b & c,** treatment groups were compared using two-way ANOVA. *P < 0.05, **P < 0.01, ***P < 0.001, and ****P < 0.0001.

We next sought to determine whether this IFN response might have originated from the dying tumor cells. Strikingly, we detected production of IFN-β and ISGs in necroptotic cells, but not apoptotic or DKO cells that lack Casp8 and MLKL. (Fig. 7a). Moreover, NEC immunization in *Ifnar1*^−/−^ mice failed to improve tumor control (Fig. 7b). Furthermore, IFNAR neutralizing antibody also abrogated the NEC immunization-mediated protection (Fig. 7c). Consistent with the notion that CD4^+^ T cells are critical for NEC immunization-mediated tumor control, CD4^+^ T cell infiltration was reduced in *Ifnar1*^−/−^ mice compared to WT controls (Fig. 7d). By contrast, NEC-induced tumor suppression and CD4^+^ T cell infiltration was comparable between WT and *Ifngr*^−/−^ mice **(Supplemental Fig. 5)**. These data suggest that necroptosis stimulates cell–intrinsic IFN-β production to initiate a cascade of reaction that triggers host type I IFN signaling to bolster anti-tumor CD4^+^ T cell responses.

## Discussion

RIPK3 signaling can stimulate necroptosis, apoptosis, and death-independent inflammatory cytokine production. To further complicate matters, DAMPs release from dying cells can also promote inflammatory gene expression. The difficulty in separating these diverse signaling events has led to conflicting reports on the role of RIPK3 signaling in anti-tumor immunity. Here, we utilized a system to drive RIPK3-dependent necroptosis or apoptosis without strong death-independent cytokine expression to interrogate the impact of necroptosis-associated DAMPs release in tumor immunity. Using this system, we observed that prophylactic immunization with necroptotic cells was sufficient to drive protective antitumor CD4^+^ T cell responses. Although both CD8^+^ and CD4^+^ T cell infiltration was enhanced, depletion experiments revealed that only CD4^+^ T cells were indispensable for this protective effect. The enhanced recruitment of CD4^+^ T cells is dependent on host type I interferon signaling, consistent with the well-known role of interferon in anti-tumor response.

Our results differ from several previous studies in which chemical-induced dimerization of chimeric RIPK3 fusion proteins led to necroptosis and concomitant RIPK1-dependent cytokine expression (21–23). The lack of RIPK1 engagement and cytokine expression in our system might be due to the use of native RIPK3 rather than RIPK3 fusion cassettes, which causes a lower level of RIPK3 nucleation. Due to the natural turnover of RIPK3, proteasome inhibition is required to elicit cell death and to unleash full protective antitumor immune responses. Since our system does not require expression of a foreign dimerization cassette, it also avoids issues such as immune reaction against exogenously introduced protein antigens.

Our results differ from the traditional view that ICD mainly stimulates DC cross-priming of CD8^+^ T cells to promote tumor protection (27, 28). CD4^+^ T cells have been described to contribute to anti-tumor immunity through a variety of mechanisms including direct killing of tumor cells, augmenting the tumor microenvironment through local secretion of effector cytokines, and providing help to CD8^+^ T cells (29–33). Our study complement prior reports that necroptotic signaling in tumors (34) and cardiac allografts (35) can bolster effector CD4^+^ T cell responses. Thus, different methods of ICD induction can elicit distinct mechanisms to confer anti-tumor immunity.

Consistent with the importance of type I IFN in anti-tumor immunity (24, 36, 37), type I IFN signaling was also critical for the protection conferred by necroptosis immunization. This type I IFN response likely originates from the necroptotic cells in a cell death-dependent manner, since the modest induction of IFNβ and ISGs was abrogated when cell death was inhibited by *Mlkl* inactivation. How might necroptosis promote this interferon response? Recent reports have shown that mitochondrial DNA (mtDNA) accumulates in the cytosol when necroptosis was induced in tumors in response to irradiation and Cisplatin (36, 37). The release of cytosolic mtDNA instigated tumor-intrinsic production of IFNβ and ISGs via the cGAS/STING pathway (36, 37). In this regard, it is noteworthy that MLKL can translocate to the nuclear and mitochondrial membranes during necroptosis (38, 39). It is tempting to speculate that MLKL-dependent pore formation may facilitate release of mtDNA to stimulate cGAS/STING, which in turn induces the first wave of IFN within the necroptotic tumor cells. However, the precise role of MLKL in mediating these effects remains to be elucidated. Further, since type I IFN signaling in the host is also required for protection mediated by NEC immunization, our data support a model in which the initial wave of IFN signal continues to propagate in the host after clearance of dying necroptotic cells to achieve optimal anti-tumor effects. However, the cellular mediators of this host response remain an open question. Nevertheless, our data suggests that, cell-intrinsic type I interferon signaling plays a key role in promoting the immunologic consequences of necroptosis.

## Methods

### Cell Lines

The LLC-OVA murine lung carcinoma cell line was generated as previously described (40). LLC-OVA, B16-F1, and human embryonic kidney (HEK) 293T cells were maintained in Dulbecco’s Modified Eagle Medium (DMEM) supplemented with 10% fetal bovine serum (FBS), 2 mM L-glutamine, and 1% penicillin-streptomycin (complete DMEM). Transduced LLC-OVA cells were maintained in complete DMEM with 2 μg/ml puromycin. All cells were cultured at 37°C with 5% CO_2_.

### Lentiviral transduction and CRISPR-Cas9 gene editing

Lentivirus was generated through transfection of HEK 293 T Cells with packaging plasmids (pMD2.G and psPAX2 vectors) and previously described plasmids containing mouse wild type *Ripk3* on a modified lentiviral tet-on pTRIPZ/Puro vector using the TransIT^®^-Lenti transfection reagent (Mirus Bio, Madison, WI, USA) (3). After 48 hours, the culture supernatant was filtered with 0.45 μm cellulose acetate filters (VWR, Radnor, PA, USA) to collect RIPK3-encoding lentivirus. LLC-OVA cells were incubated with lentivirus in complete DMEM containing polybrene (8 μg/mL) for an additional 48 hours. Transduced cells were selected with 2 μg/ml puromycin.

For generating RIPK1, CASP8 and MLKL-deficient cell lines, the following guide RNA (gRNA) sequences were cloned into LentiCRISPRv2-Blast lentiviral vector [a gift from Mohan Babu; Addgene plasmid # 83480]: 5’- CAGACTGAGACACAGTCGAG-3’ (murine *Ripk1* gRNA #1), 5’- TGTGAAAGTCACGATCAACG-3’ (murine *Ripk1* gRNA #2), 5’-AGACGACACCCTTGTCACCG-3’ (murine *Casp8* gRNA #1), 5’- AGATGTCAGGTCATAGATGG-3’ (murine *Casp8* gRNA #2), 5’-CAAAGTATTCAACAACCCCC-3’ (murine *Mlkl* gRNA #1), 5’- AGGAACATCTTGGACCTCCG-3’ (murine *Mlkl* gRNA #2). Constructs were transduced into LLC OVA cells as described above and selected in Blasticidin (8 μg/mL).

### Mice

Age- and sex-matched mice of C57BL/6J background were used for these experiments unless otherwise specified. C57BL/6J (Stock No: 000664), OT-II (B6.Cg-Tg(TcraTcrb)425Cbn/J, Stock No: 004194), CD45.1 (B6.SJL-*Ptprc^a^Pepc^b^*/BoyJ, Stock No: 002014), and IFN-αR^−/−^ (B6(Cg)-Ifnar1^tm1.2Ees/^J, Stock No: 028288), mice were purchased from The Jackson Laboratory (Bar Harbor, ME, USA). *Batf3*^−/−^ mice (B6.129S(C)-*Batf3^tm1Kmm^*/J) were kindly provided by Dr. Dee Gunn (Stock No: 013755). All mice were housed in a specific pathogen-free (SPF) facility at Duke University and maintained according to protocols approved by the Duke University Institutional Animal Care and Use Committee.

#### In vivo prophylactic dying tumor cell immunization

Tumor cells were seeded on 15-cm tissue culture dishes and cell death was induced *in vitro* by treating cells with DOX (1 μg/ml) for 9 hours followed by MG132 (4 μM, APExBio, Houston, TX, USA) for 4.5 hours. Dying tumor cells were then collected, washed twice in PBS (Thermo Fisher Scientific, Waltham, MA, USA), then re-suspended at 7.5 × 10^6^ cells/mL in PBS. Mice were immunized subcutaneously with 7.5 × 10^5^ cells (100 μl) in the right flank. On day 8 after vaccination, mice were challenged subcutaneously on the left flank with 5 × 10^5^ live tumor cells suspended in serum free-DMEM mixed 1:1 with Matrigel (Matrigel^®^ Basement Membrane Matrix, LDEV-free, Corning Life Sciences, Tewksbury, MA, USA). Tumor growth on the challenge site was evaluated using calipers. Tumor volume was calculated using the formula: 0.5 × long axis × short axis^2^. Mice were euthanized if tumors exceeded 2000 mm^3^.

#### In vivo antibody administration

For INFAR1 blockade, 1 mg of anti-IFNAR-1 antibody (clone MAR1–5A3, Bio X Cell, Lebanon, NH, USA) or isotype control was administered to mice intravenously via retroorbital injection the day prior to dying cell immunization. For T cell depletion, 350 μg of anti-CD8 (clone YTS 169.4, Bio X Cell), anti-CD4 (clone GK1.5, Bio X Cell), or Isotype control was administered to mice intravenously prior to dying cell immunization. Where indicated, an additional 150 μg of anti-CD8, anti-CD4, or Isotype control was administered on the day prior to dying cell immunization.

### OT-II adoptive transfer

For OT-II adoptive transfer experiments, spleens were collected from congenic OT-II mice, mechanically homogenized and filtered through a 70 μM cell strainer. Erythrocytes were then lysed using ACK Lysis Buffer (150 mM NH_4_Cl, 10 mM KHCO_3_, 0.1 mM Na_2_EDTA). Splenocytes were subsequently counted and the percentage of CD4^+^ T cells was determined by flow cytometry. Splenocytes were resuspended in RPMI at 10 × 10^6^ CD4^+^ T cells/mL and 100 μl was administered to mice intravenously via retroorbital injection.

### Splenocyte co-culture with necroptotic cells

For expansion of endogenous myeloid populations *in vivo*, mice were implanted subcutaneously on the flank with 2.5 × 10^5^ cells Flt3L expressing B16 cells (B16-Flt3L) (41). On day 14 post-tumor implantation, spleens were collected, minced and digested in HBSS with Ca and Mg (Thermo Fisher Scientific) + 5% FBS + 10mM HEPES + 2 mg/mL Type IV Collagenase (Sigma, St. Louis, MO, USA C-5138) + 10 IU/ml DNase I (Sigma D4263–1VL) for 30 minutes at 37°C. Spleens were then homogenized and filtered through a 70 μM cell strainer. Erythrocytes were then lysed using ACK Lysis Buffer (150 mM NH_4_Cl, 10 mM KHCO_3_, 0.1 mM Na_2_EDTA).

Cell death was induced in tumor cells with DOX (1 μg/ml) for 9 hours followed by MG132 (4 μM, APExBio) for 4.5 hours. Dying cells were collected for co-culture. Dying tumor cells and splenocytes were co-cultured at a 10:1 ratio in a 24 well plate with 0.5 mL of RPMI 1640 with 10% fetal bovine serum (FBS), 1% Non-essential Amino Acids, 1% sodium pyruvate, 2 mM L-glutamine, and 1% penicillin-streptomycin. Cells were harvested 8 and 24 hours later for flow cytometry analysis.

### Flow cytometry

Single cell suspensions were obtained from tumors by digesting minced tumor tissue in complete RPMI containing type IV collagenase (1 mg/ml, Sigma C-5138) and deoxyribonuclease I (20 IU/ml, Sigma D4263–1VL) at 37°C with gentle agitation for 30 minutes followed by tissue homogenization. Cell suspension was then passed through a 70 μM cell strainer. Erythrocytes were lysed using ACK Lysis Buffer. Two million cells were stained with LIVE/DEAD fixable aqua dead cell stain kit (Thermo Fisher Scientific) for 30 mins at 4°C. Cells were incubated with Fc-blocking antibody (clone 2.4G2) for 15 mins prior to incubation with fluorochrome-conjugated antibodies in 1x PBS, 2% FBS, and 2 mM EDTA at 4°C for 30 mins. Flow cytometry was performed on a BD Fortessa instrument. Analysis of flow cytometry data was done using FlowJo Treestar) software (version 10.8.1).

Cells were stained with the following antibodies: NK1.1 (PK136, FITC), CD11b (M1/70, PerCP-Cy5.5), CD11b (M1/70, PE-Cy7), CD19 (6D5, PE-Cy7), CD3 (17A2, APC), CD3 (17A2, FITC), I-A/I-E (M5/114.15.2, AlexaFluor 700), CD8β (YTS156.7.7, APC-Cy7), CD45–2 (104, Pacific Blue, CD45–2 (30-F11, BV605), CD45–1 (A20, FITC), Ly6C (HK1.4, BV605), B220 (RA3–6B2, BV650), XCR1 (ZET, BV785), CD11c (N418, PE), CD4 (GK1.5, PE-Cy5), Ly6G (1A8, PE-Dazzle594), F4/80 (BM8, PE-Cy7, Sirpα (P84, APC), CD80 (16–10A1, PE-Dazzle594), CD44 IM7, BV711), CD62L (MEL-14, PE), PD-1 (29F.1A12, PE-Cy7) from Biolegend (San Diego, CA, USA) and TCR-β (H57–597, APC) from eBiosciences (San Diego, CA, USA).

### NanoString RNA analysis and qRT-PCR

To assess tumor cell cytokine production, cell death was induced in tumor cells using DOX and MG132. Total RNA was isolated using the Qiagen RNeasy Mini Kit (Qiagen). For Nanostring analysis, RNA was run on a NanoString nCounter Pro Analysis System using an nCounter Mouse Tumor Signaling 360 Panel (Nanostring, Seattle, WA, USA). Data were normalized and analyzed using ROSALIND software (NanoString). We thank the Duke University School of Medicine for the use of the Microbiome Core Facility, which provided NanoString Gene Expression service. For qPCR, cDNA was synthesized using the iScripts cDNA synthesis kit (Bio-Rad 170–8891). Thermal cycling reaction was then performed using iQ^™^ SYBR^®^ Green Supermix (Bio-Rad, Hercules, CA, USA 170–8882) and a CFX Connect Real-Time PCR Detection System (Bio-Rad). Cycle threshold (CT) values for target genes were normalized to CT values of the housekeeping gene *Tbp1* (ΔCT = CT(Target) − CT(Tbp1)) and subsequently normalized to baseline control values (ΔΔCT = ΔCT(Experimental) − ΔCT(Control)).

#### Primers:

**Table:** 

*Mtbp*	Forward	CAAACCCAGAATTGTTCTCCTT
	Reverse	ATGTGGTCTTCCTGAATCCCT
*Tnf*	Forward	CCCACTCTGACCCCTTTACT
	Reverse	TTTGAGTCCTTGATGGTGGT
*Ccl2*	Forward	AGGTGTCCCAAAGAAGCTGTA
	Reverse	ATGTCTGGACCCATTCCTTCT
*Cxcl1*	Forward	CGAAGTCATAGCCACACTCAA
	Reverse	GAGCAGTCTGTCTTCTTTCTCC
*Ifnb1*	Forward	AATTTCTCCAGCACTGGGTG
	Reverse	AGTTGAGGACATCTCCCACG
*Ifit1*	Forward	CACCAGTATGAAGAAGCAGAGAG
	Reverse	GCCATAGCGGAGGTGAATATC
*Ifi44*	Forward	GGGCTGTGATGAAGATGGAA
	Reverse	CCCAGTGAGTCACACAGAATAA
*Ifi208*	Forward	GCACAGAGAAGAGAAGGAGAAA
	Reverse	CTGTTGTCTGTGGTGGAGATAG
*Ifi213*	Forward	GATGGAAGCTTGGGAAGTAGAA
	Reverse	GAGAGAACGAGCTTAGTGGATG
*Tgtp1*	Forward	CTTCCCAAAGCTGGAAACTAAAC
	Reverse	GTTAATGGTGGCCTCAGTAAGA
*Tgpt2*	Forward	CTTCCCAAAGCTGGAAACTAAAC
	Reverse	GTTAATGGTGGCCTCAGTAAGA

### RNAseq

Total RNA was extracted from single cell suspensions from tumor tissues. A mRNA library was prepared using the DNBSEQ platform by BGI with data filtering using the SOAPnuke software (42). HISAT2 was selected to map the filtered sequenced reads to the reference genome. BAM files containing mapping results were counted using the featureCounts function using Python. Counting was performed using the mouse genome for comparison. Downstream analyses were performed using iDEP.96 web interface (43). DEG analysis was then performed using DESeq2 considering all genes with FDR ≤ 0.1 and 1 ≤ Log2FC ≤ −1. Functional analysis of genes with FDR ≤ 0.1, regardless of Log2FC, comprised of GO and GSEA (Gene Set Enrichment Analysis) analyses. For GSEA, gene sets used in this assessment included curated gene sets, known pathways (KEGG), and gene ontology terms (Biological Process & Molecular Function).

### Incucyte Cell Death Assays

Cells were seeded in a 96-well plate with 10,000 cells per well in 200 μl complete growth medium. Eight hours prior to cell death initiation, medium was exchanged for Complete DMEM with DOX (1 μg/ml) or DMSO. Cells were subsequently treated with MG132 (4 μM, APExBio). Imaging was subsequently performed using the IncuCyte S3 (Sartorius, Göttingen, Germany; version 2021C). Nine images per well were captured, analyzed, and averaged. Cell death was assessed through measuring uptake of YoYo-1 (50 nM, Thermo Fisher Scientific) and expressed as the area of YoYo-1^+^ cells as a percentage of the total phase area. In experiments where zVAD-fmk and GSK’843 were used, zVAD-fmk (20 μM, APExBio) was administered 30 minutes prior to treatment with GSK’843 (20 μM, Sigma) and MG132.

### Western blot

Cell lysates were prepared in RIPA buffer containing 0.15 M NaCl, 0.05 M Tris (pH 8.0), 0.1% SDS, 0.5% Sodium deoxycholate, and 1% Nonidet P-40 supplemented with Protease (Roche, Basel, Switzerland11836145001) and Phosphatase inhibitor cocktails (Sigma P5726). Protein concentration was determined using a BCA Protein Assay (Thermo Fisher Scientific). The proteins were separated by SDS-PAGE and transferred to nitrocellulose membranes. Primary antibodies used were anti-MLKL phospho-S345 (Cell Signaling Technology, Danvers, MA, USA 37333), anti-MLKL (Cell Signaling Technology, 37705), anti-RIPK3 phospho-S232 (Abcam, Cambridge, United Kingdom, ab195117), anti-RIPK3 (Genentech, San Francisco, CA, USA, PUR135347), anti-RIPK3 (Prosci, Poway, CA, USA, 2283), anti-RIPK1 (BD Biosciences, Franklin Lakes, NJ, USA, 610459), anti-cleaved caspase-3 (Cell Signaling Technology, 9664), anti-caspase-8 (Enzo, Farmingdale, NY, USA, ALX-804-447-C100), anti-p65 phospho-S536 (Cell Signaling Technology, 3033), anti-p65 (Santa Cruz Biotechnology, Dallas, TX, USA sc-8008), anti-IκBα phospho-S32/36 (Cell Signaling Technology, 9246), anti-IκBα (Cell Signaling Technology, 4814), anti-Actin (Cell Signaling Technology, 3700). HRP-conjugated goat anti-rabbit immunoglobulin G (IgG) (111-035-144), rabbit anti-mouse IgG (315-035-008) or goat anti-rat IgG (112-035-175) were obtained from Jackson ImmunoResearch Laboratories Inc (West Grove, PA, USA). After incubation with the appropriate secondary antibodies, membranes were incubated with Clarity ECL western blotting substrate (Bio-Rad, 170–5061) or Clarity Max ECL (Bio-Rad, 170–5062).

### Statistics

Statistical analysis was performed in GraphPad Prism (version 9). Unpaired two-tailed Student’s t-test was used to compare two independent groups. Tukey’s multiple comparison test, or one-way Analysis of variance (ANOVA) or two-way ANOVA were used to compare multiple (> 2) groups with one or two independent variables, respectively; with multiple comparisons tests as indicated. P values > 0.05 were considered statistically non-significant. *p value < 0.05, **p value < 0.01, ***p value < 0.001, ****p value < 0.0001.
